# The critical role of endothelial function in fine particulate matter-induced atherosclerosis

**DOI:** 10.1186/s12989-020-00391-x

**Published:** 2020-12-04

**Authors:** Shuang Liang, Jingyi Zhang, Ruihong Ning, Zhou Du, Jiangyan Liu, Joe Werelagi Batibawa, Junchao Duan, Zhiwei Sun

**Affiliations:** 1 Department of Toxicology and Sanitary Chemistry, School of Public Health, Capital Medical University, Beijing, 100069 People’s Republic of China; 2 Beijing Key Laboratory of Environmental Toxicology, Capital Medical University, Beijing, 100069 People’s Republic of China

**Keywords:** PM_2.5_, Endothelial dysfunction, Inflammation, Coagulation, Lipid deposition, Atherosclerosis

## Abstract

Ambient and indoor air pollution contributes annually to approximately seven million premature deaths. Air pollution is a complex mixture of gaseous and particulate materials. In particular, fine particulate matter (PM_2.5_) plays a major mortality risk factor particularly on cardiovascular diseases through mechanisms of atherosclerosis, thrombosis and inflammation. A review on the PM_2.5_-induced atherosclerosis is needed to better understand the involved mechanisms. In this review, we summarized epidemiology and animal studies of PM_2.5_-induced atherosclerosis. Vascular endothelial injury is a critical early predictor of atherosclerosis. The evidence of mechanisms of PM_2.5_-induced atherosclerosis supports effects on vascular function. Thus, we summarized the main mechanisms of PM_2.5_-triggered vascular endothelial injury, which mainly involved three aspects, including vascular endothelial permeability, vasomotor function and vascular reparative capacity. Then we reviewed the relationship between PM_2.5_-induced endothelial injury and atherosclerosis. PM_2.5_-induced endothelial injury associated with inflammation, pro-coagulation and lipid deposition. Although the evidence of PM_2.5_-induced atherosclerosis is undergoing continual refinement, the mechanisms of PM_2.5_-triggered atherosclerosis are still limited, especially indoor PM_2.5_. Subsequent efforts of researchers are needed to improve the understanding of PM_2.5_ and atherosclerosis. Preventing or avoiding PM_2.5_-induced endothelial damage may greatly reduce the occurrence and development of atherosclerosis.

## Background

The World Health Organization (WHO) reported that approximately 91% of people worldwide live in unhealthy environments where air quality levels exceed WHO limits. The combined effects of indoor and ambient air pollution result in approximately 7 million premature deaths from noncommunicable diseases every year [1]. Chemicals in the air initiate or potentiate a wide range of noncommunicable diseases [2]. Fine particulate matter (PM_2.5_, the aerodynamic diameter ≤ 2.5 μm) in air pollution became the fifth death risk factor in 2015 [3]. PM_2.5_ is a complex mixture, and its major source is combustion, such as traffic-related diesel exhaust particles (DEPs), industry, indoor cooking activities, and bushfires [4]. For example, the Australian bushfires in 2019-2020 had extreme impacts on air quality throughout the region and even the globally [5]. Thus, the global burden of cardiovascular disease caused by PM_2.5_ may be much greater than that previously reported by WHO. Evidence has indicated that PM_2.5_ induces lipid metabolism dysregulation and increases hypertension and the prevalence of cardiac arrhythmias, thus accelerating the progression of atherosclerosis, and increasing the risk of cardiovascular disease- and stroke-related mortality [6–9].

Atherosclerosis is a chronic inflammatory disease of large and medium-sized arteries. The causes of atherosclerosis are inflammation, hemodynamic damage and abnormal lipid metabolism in early-stage atherosclerosis [10]. When endothelial cells are activated, they express inflammatory cytokines (such as interleukin (IL)-8, monocyte chemoattractant protein (MCP)-1) and adhesion molecules (such as intercellular adhesion molecule (ICAM-1) and vascular cell adhesion molecule (VCAM-1)), attracting blood monocytes that bind to the activated endothelial monolayer and infiltrate the arterial wall. Important biomarkers of the development of atherosclerotic inflammation include C-reactive protein (CRP), IL-6, adhesion molecules, and matrix metalloproteinases (MMPs) [10]. These factors induce macrophage polarization into the pro-inflammatory M1-like or anti-inflammatory M2-like phenotype [11]. The scavenger receptors of macrophages, such as low-density lipoprotein receptor-related protein 1 (LRP1), cluster of differentiation 36 (CD36) and class B type 1 (SR-B1), play a key role in lipid uptake, deposition and the development of atherosclerotic plaques [12–14]. Liver X receptor α (LXR-α)/ATP-binding cassette transporter A1 (ABCA1)/ABCG1-dependent cholesterol efflux is a crucial event in the suppression of lipid accumulation during the transformation of macrophage foam cells [15]. Vascular smooth muscle cells (VSMCs) migrate from the media to the intima, synthesize extracellular matrix macromolecules such as elastin, proteoglycans and collagen, and form fibrous caps formation. The death of foam cells and VSMCs leads to the release of extracellular lipids in atherosclerotic lesions, leading to the formation of a necrotic core [11, 16]. MMPs (such as MMP-9) are highly expressed in atherosclerotic plaques, leading to substantial enhancement of elastin degradation and inducing plaque rupture [17]. Currently, several imaging techniques can be used to investigate plaques and signs of vulnerability, such as CT, magnetic resonance imaging (MRI) and ultrasound [18]. Molecular imaging is an innovative technique for the detection of plaque inflammation. The utility of several nanoparticles, such as sodium fluoride, iron oxide and polyethylene glycol molecules, for the molecular imaging of atherosclerosis in animal models and patients has been investigated [19–21]. In the past few decades, treatment strategies for atherosclerosis have mainly focused on lowering lipid levels with high-intensity statins. However, only approximately 25% of patients who receive high-intensity statins as a lipid-lowering therapy achieve the recommended level of low-density lipoprotein cholesterol (LDL-C, ≤ 1.8 mmol/L) [22]. Approximately 75% of patients do not respond to statin therapy sufficiently; therefore, novel therapeutic strategies are needed.

PM_2.5_ is a complex mixture, and a review had comprehensively summarized the chemical composition and characteristics of PM_2.5_, including inorganic elements, water-soluble ions, carbonaceous aerosols and organic compounds (polycyclic aromatic hydrocarbons (PAHs) and volatile organic compounds (VOCs) etc) [4].. A study showed that coal combustion and vehicular emissions are the main sources of PAHs and VOCs in PM_2.5_ [23, 24]. Evidence has demonstrated that DEPs accelerate the development or exacerbation of atherosclerosis [25, 26]. Organic chemicals from DEPs, such as PAHs adhere to the carbon cores of particles, and certain PAHs can trigger Ca^2+^ signaling and increase inflammation in endothelial cells [27–30]. Evidence has shown that the levels of urinary PAH biomarkers are associated with cardiovascular disease [31]. Due to the antagonistic and synergic effects of complex VOC mixtures, the toxic effects of VOCs are difficult to estimate [32]. In addition, the surface of particles may bind reactive copollutants, including biomolecules (such as endotoxins), redox-active transition metals, and reactive quinones/aldehydes, which may be carried by particles and enter lung tissue and the circulation, inducing secondary toxicity [33, 34]. The ions and metal components of PM_2.5_, including SO_4_^2−^, K^+^, Cl^−^, K, Si, As, Zn, Se and Pb, could be mainly responsible for systemic inflammation [35]. Evidence has shown that the binding of endotoxins to the surface of PM_2.5_ particles plays a critical role in the inflammatory response. Endotoxin neutralizer (polymyxin B) and knockout of toll-like receptor 4 (TLR4) strongly inhibit the PM_2.5_-triggered inflammatory response [36]. However, the understanding of the major toxic effects exerted by the specific components of PM_2.5_ is limited. Further investigating the toxicity of PM_2.5_ components will contribute to a comprehensive understanding of PM_2.5_, which may be a key area of future research.

Endothelial cells cover the internal surface of blood vessels, and the integral endothelial cell layer maintains a complex functional balance to inhibit the inflammatory response or thrombosis. Ambient PM_2.5_ exposure elicits the deterioration of endothelial function, systemic inflammation and coagulation [37, 38]. Evidence has shown that a 10 μg/m^3^ increase in the PM_2.5_ concentration at a 1-day lag was associated with increased brachial-ankle pulse wave velocity (baPWV, a physiological indicator of arterial stiffness), but not with high-sensitivity C-reactive protein (hsCRP, a biomarker of vascular inflammation) levels; thus, arterial stiffness might be more sensitive to ambient PM_2.5_ exposure than inflammation [39]. Accordingly, indoor PM_2.5_ also induces endothelial dysfunction and inhibits blood vessel formation but has no significant association with arterial stiffness [40, 41]. Endothelial dysfunction disrupts anti-inflammatory processes, anti-platelet aggregation, anti-thrombotic processes and vascular repair *in vivo* [42]. Alterations in vascular function might be the earliest pathophysiological mechanism contributing to air pollution-mediated cardiovascular diseases, and indeed, such changes are a critical early predictor of atherosclerosis [43, 44]. However, there is a lack of systematic understanding of the mechanism of PM_2.5_-induced endothelial dysfunction. Moreover, a review focused on scientific evidence that DEPs induce endothelial dysfunction, including bioavailability and mechanisms, and is related to cardiovascular injury [45].

**Fig. 1:**
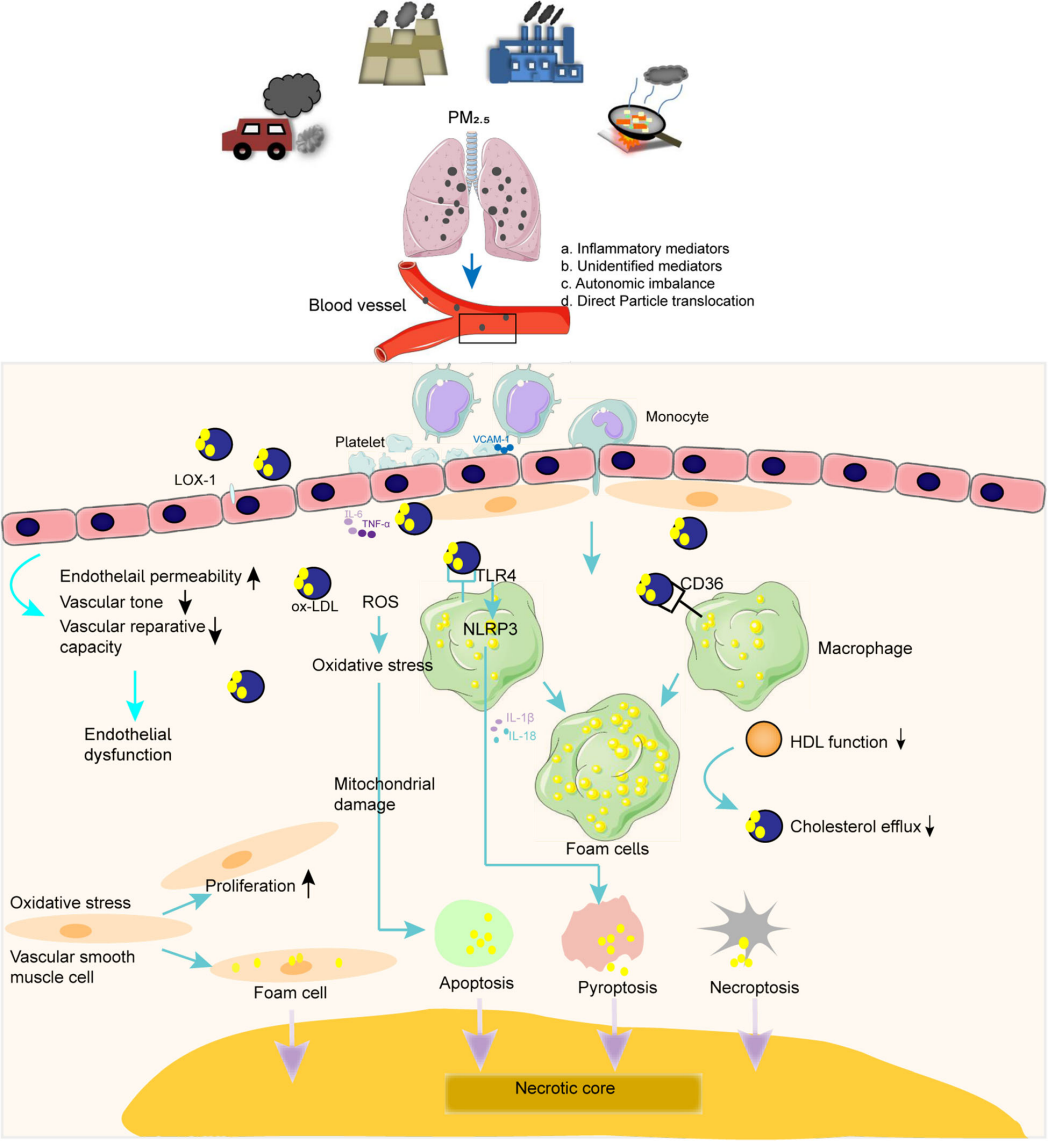
Summarized the main pathogenic mechanisms of PM_2.5_-triggered atherosclerosis. Four main hypotheses have proposed by which inhaled particulate matter effect on cardiovascular system [49]: **a**. inflammatory mediators; **b**. unidentified mediators; **c**. autonomic imbalance; **d**. direct particle translocation. PM_2.5_ increased endothelial permeability, declined vascular tone and vascular reparative capacity, thus induced vascular endothelial injury. The initial step of atherosclerosis is vascular endothelial dysfunction, and then activated endothelial cells promoted monocytes recruited and maturation of monocytes into macrophages. Lipid accumulation and continued uptake by macrophages lead to foam cell formation and then developed into atherosclerotic lesion

Therefore, this review mainly provides an overview of the literature related to PM_2.5_ and atherosclerosis, and discusses the mechanism of PM_2.5_-induced vascular endothelial injury. Approximately 30% of ambient PM_2.5_ is attributable to traffic sources [46–48]. Thus, we also review evidence for the role DEPs in atherosclerosis and endothelial dysfunction in this review. Fig. 1 summarizes the main mechanisms of PM_2.5_-triggered atherosclerosis. We review the main mechanisms of endothelial dysfunction after exposure to ambient or indoor PM_2.5_. Furthermore, we discuss the role of ambient PM_2.5_-induced vascular endothelial injury in the development of atherosclerosis. Targeting endothelial injury, as the initial pathological process of atherosclerosis, is the key to preventing the occurrence of atherosclerotic cardiovascular disease. Therefore, a scientific understanding of the mechanism of PM_2.5_-induced endothelial dysfunction will play a critical role in the prevention and treatment of atherosclerosis and other cardiovascular-related diseases.

## PM_2.5_ and atherosclerosis

As shown in Fig. 1, four main hypotheses by which inhaled particulate matter affects the cardiovascular system have been proposed: a. Inhaled particulate matter reaches the terminal bronchioles and enters alveoli, inducing an inflammatory response in the lung; b. Released inflammatory mediators and unidentified mediators enter the circulation; c. A small proportion of particles reach the circulation; and d. Inhaled particulate matter activates alveoli sensory receptors, leading to autonomic imbalance [49]. Fig. 1 summarizes the main mechanisms of PM_2.5_-triggered atherosclerosis. Endothelial injury increases the release of IL-6, VCAM-1, ICAM-1, and other inflammatory cytokines, recruiting blood monocytes that bind to the activated endothelial monolayer. The bound monocytes migrated directly into the intima and mature into macrophages. PM_2.5_ increases the expression of CD36 in plaque macrophages and mediates the abnormal accumulation of oxidized lipids (such as 7-ketocholesterol, 7-KCh), finally promoting foam cell formation [12]. TLR4 recognizes modified lipoprotein, which mediates lipoprotein accumulation in macrophages [50]. Jin Geng et al. showed that PM_2.5_ can trigger foam cell formation via the TLR4/MyD88/NF-κB pathway [51]. Oxidized low-density lipoprotein (ox-LDL) primes and activates the NOD-like receptor protein 3 (NLRP3) inflammasome by binding to TLR4 or CD36 in macrophages and increases the release of inflammatory cytokines (IL-1β and IL-18) and pyroptosis [11]. PM_2.5_ induces oxidative stress, increasing the apoptosis of foam cells via the mitochondrial apoptosis pathway [52]. PM_2.5_ impairs HDL functions such as HDL-mediated cholesterol efflux, thus facilitating foam cell formation and accumulation [53]. Apoptotic cells are not quickly and efficiently engulfed and decomposed by phagocytes, resulting in secondary necrosis and the release of a large amount of pro-inflammatory cytokines and thus contributing to the development of the necrotic core [54]. However, currently, there is a lack of evidence concerning the efferocytosis of phagocytes in atherosclerosis induced by PM_2.5_. In addition, oxidative stress induced by PM_2.5_ can increase the proliferation and foam cell formation in VSMCs; however, future research is required to demonstrate the role of oxidative stress in mediating PM-triggered foam cell formation [55, 56]. VSMCs migrate from the media to the intima and synthesize extracellular matrix macromolecules such as elastin, proteoglycans and collagen, and fibrous cap formation. However, reports concerning the migration of VSMCs triggered by PM_2.5_ are lacking. The death of foam cells and the release of extracellular lipids in atherosclerotic lesions lead to the formation of a necrotic core [11, 16].

Table 1 summarizes epidemiological studies on the association between PM_2.5_ exposure and atherosclerosis. One of the major sources of ambient PM_2.5_ is traffic-derived emissions. Traffic noise is an important confounding factor in the effects of air pollutant exposure from traffic, and evidence has demonstrated that night-time traffic noise and PM_2.5_ are both associated with a 3.9% (95% CI: 0.0 - 8.0%) increase in thoracic aortic calcification (TAC)-burden per 5 dB(A) night-time traffic noise and an 18.1% (95% CI: 6.6 - 30.9%) increase in TAC burden per 2.4 μg/m^3^ PM_2.5_. Importantly, both are independently associated with the development and progression of subclinical atherosclerosis [59, 71]. Carotid intima-media thickness (CIMT) is defined as the distance between the lumen-intima and media-adventitia borders of the common carotid artery and can be measured by vascular ultrasound; an increase in the CIMT is a marker of subclinical atherosclerosis [93]. PM_2.5_ exposure is associated with an increase in the CIMT; moreover, increased or slowed CIMT progression is associated with PM_2.5_ concentration [68]. The PM_2.5_ components sulfur, elemental carbon (EC) and organic carbon (OC), but not silicon, are associated with increased CIMT, and OC has the strongest association [69, 78]. Long-term exposure to PM_2.5_ can increase systematic inflammation, the levels of fibrofatty and necrotic core components, and total plaque volume [38, 79]. Short-term exposure to PM_2.5_ is associated with inflammation, coagulation, endothelial activation and ox-LDL levels [38, 63]. Table 2 summarizes animal studies of PM_2.5_-induced atherosclerosis. PM_2.5_ promotes the progression of advanced atherosclerotic lesions. Concentrated ambient PM_2.5_, rather than whole diesel exhaust and diesel exhaust gases, is mainly responsible for plaque exacerbation [99]. However, an improved understanding of which components of PM_2.5_ induce or promote the development of atherosclerosis, which may be a direction of future research, is needed. Mechanistically, changes in oxidative stress, systematic inflammation and lipid metabolism are the most common mechanisms of PM_2.5_-induced atherosclerosis. The mechanisms of atherosclerosis induced by PM_2.5_ have been reviewed [105]. Oxidative stress induced by particulate matter may be key to triggering the development of atherosclerosis, coagulation and thrombosis [106, 107]. Endothelial dysfunction is a critical initiating event that promotes the development of atherosclerosis. However, a systematic understanding of the mechanisms underlying PM_2.5_-induced endothelial dysfunction leading to atherosclerosis is lacking.

**Table 1:** Epidemiological studies on the association between PM_2.5_ exposure and atherosclerosis

Reference	Location	Study design	Sample size	Pollutants	PM_2.5_ Exposure	Evaluation index	Findings or association
[57]	-	Meta-analysis	9183	Ambient PM_2.5_, PM_10_, PM_2.5abs_, PMcoarse, NOx, NO_2_	-	CIMT	PM_2.5_ (per 5 μg/m^3^ increase): CIMT increased by 0.78% (95% CI: -0.18%, 1.75%, p = 0.11).
[58]	Ohio, United States	Prospective longitudinal cohort	6575	Ambient PM_2.5_, NO_2_	Long-term exposure	Angiography	PM_2.5_ (per 2.2 μg/m^3^ increase): Mild coronary atherosclerosis (defined as 1 to 2 vessels with ≥ 50% stenosis) OR = 1.43 (95% CI: 1.11-1.83; p = 0.005); Severe coronary atherosclerosis (defined as 3 vessels with ≥ 50% stenosis) OR = 1.63 (95% CI: 1.26 to 2.11; p < 0.001).
[59]	CA, USA	Cross-sectional	4238	PM_2.5_, traffic noise	Long-term exposure	TAC	PM_2.5_ (per 2.4 μg/m^3^ increase): TAC burden increased by 18.1% (95% CI: 6.6 to 30.9%).
[60]	USA	Longitudinal cohort	6814	Ambient PM_2.5_ NOx, NO_2_ and black carbon	Long-term exposure	CAC; IMT	PM_2.5_ (per 5 μg/m^3^ increase): Coronary calcium progressed by 4.1 Agatson units per year (95% CI: 1.4 to 6.8); Without association with IMT, -0.9 μm per year (95% CI: -3.0 to 1.3).
[61]	India	prospective, intergenerational cohort	3278	Ambient and indoor air pollution	Long-term exposure	CIMT	Ambient PM_2.5_ (per 1 μg/m^3^ increase): CIMT increased by 1.79% (95% CI: -0.31 to 3.90) in all participants; CIMT increased by 2.98% (95% CI: 0.23 to 5.72) in men. Indoor air pollution (biomass cooking fuel): CIMT increased by 1.60% (95% CI: -0.46 to 3.65) in all participants
[62]	-	Meta-analysis	-	PM_2.5_	-	CIMT arterial calcification; ankle-brachial index	PM_2.5_ (per 10 μg/m^3^ increase): CIMT increased by 22.52 μm (p = 0.06); Without association with arterial calcification (p = 0.44) or ankle-brachial index (p = 0.85).
[63]	USA	Cross-sectional	6654	Ambient PM_2.5_ and black carbon	12 months, 3 months 2 weeks Short-term exposure (0-5 days)	HDL-C HDL particle number	No significant association between PM_2.5_ and HDL-C; PM_2.5_ (per 5 μg/m^3^ increase) exposure for 3 months: HDL-P decreased by 0.64 μmol/L (95% CI: -1.01 to -0.26); PM_2.5_ (per 5 μg/m^3^ increase) exposure for 2-week: HDL-C increased by -0.86 mg/dL (95% CI: -1.38 to -0.34); HDL-P decreased by 0.29 μmol/L (95% CI: -0.57 to -0.01). PM_2.5_ (per 5 μg/m^3^ increase) exposure for 5 days: HDL-P decreased by 0.21 μmol/L (95% CI: -0.38 to -0.04).
[64]	Beijing, China	Panel study	40	Ambient PM_2.5_	Short-term exposure (1 day)	Ox-LDL; sCD36	PM_2.5_ chloride, strontium, iron (1-day, per 0.51 μg/m^3^ increase) and nickel (2-day, 2.5 μg/m^3^ increase): ox-LDL increased by 1.9% (95% CI: 0.2% to 3.7%, p < 0.05) and 1.8% (95% CI: 0.2% to 3.4%), respectively; PM_2.5_ calcium (1-day, 0.7 μg/m^3^ increase): sCD36 increased by 4.8% (95% CI: 0.7% to 9.1%).
[65]	Beijing, China	Cross-sectional	8867	Ambient PM_2.5_, NO_2_, O_3_	Long-term exposure	CAC Score	PM_2.5_ (per 30 μg/m^3^ increase): CAC scores increased by 27.2% (95% CI: 10.8% to 46.1%); CAC increased by 42.2% (95% CI: 24.3% to 62.7%) in men, 50.1% (95% CI: 28.8% to 75%) in elderly participants, 62.2% (95% CI: -1.4% to 20.4%) in those with diabetes.
[66]	Taiwan	Cross-Sectional	689	Ambient PM_10_, PM_2.5_, PM_2.5_abs, NO_2_, NOx	Long-term exposure	CIMT	PM_2.5_abs (per 1.0 x 10^-5^/m): Maximum left CIMT increased by 4.23% (95% CI: 0.32% to 8.13%, p < 0.05); PM_2.5_ mass concentration was not associated with CIMT.
[67]	Toronto	Cohort study	30	Urban PM_2.5_ and O_3_	Short-term exposure (2 h)	HOI; Blood pressure;	PM_2.5_ (exposure for 2h, 1h after exposure): Association with HOI (p = 0.078); HOI associated with systolic blood pressure (p = 0.05).
[68]	USA	Cross-sectional, longitudinal	5276	PM_2.5_	Long-term exposure	CIMT	PM_2.5_ concentration (per 2.5 μg/m^3^ increase): Increased IMT progression (5.0 μm/y, 95% CI: 2.6 to 7.4 μm/y); PM_2.5_ concentration (per 1 μg/m^3^ reduce): Slowed IMT progression (-2.8 μm/y, 95%CI: -1.6 to -3.9μm/y).
[69]	USA	Cross-sectional	5488	Ambient PM_2.5_	Long-term exposure	CIMT	PM_2.5_ (sulfur, silicon, EC and OC): Association: CIMT Sulfur (0.022 mm, 95% CI: 0.014 to 0.031); silicon (0.006 mm, 95% CI: 0.000 to 0.012); OC (0.026 mm, 95% CI: 0.019 to 0.034).
[70]	South India	Cross-sectional	7000	PM_2.5_	-	CIMT	PM_2.5_ (per 1 μg/m^3^ increase): Association: CIMT.
[71]	Germany	Cohort study	4814	Traffic- related air pollution and noise	Long-term exposure	TAC	No associations between PM_2.5_ and TAC
[72]	USA	Longitudinal	165675	Ambient PM (PM_10_, PM_2.5_, PM_2.5-10_)	Long-term exposure; Short-term exposure	Leukocyte Counts and Composition	PM_2.5_ (per 10 μg/m^3^ increase, exposure for 1-month): Increased: leukocyte count (12 cells/μl, 95%CI: -9 to 33), granulocyte proportion (1.2%, 95% CI: 0.6% to 1.8%); Decreased: CD8^+^ T cell (-1.1%, 95%CI: -1.9% to -0.3%); PM_2.5_ (per 10 μg/m^3^ increase, exposure for 12-month): Increased: leukocyte count (28 cells/μl, 95%CI: -20 to 75), granulocyte proportion (1.1%, 95% CI: -0.2% to 2.4%); Decreased: CD8^+^ T cell (-1.3%, 95%CI: -2.4% to -0.1%);
[38]	USA	Longitudinal	6814	Ambient PM_2.5_	Long-term exposure; Short-term exposure	Serum CRP, IL-6, fibrinogen, D-dimer, soluble E-selectin, sICAM -1	Long-term exposure to PM_2.5_ ( per 10 μg/m^3^ increase): Association: inflammation and fibrinolysis (CRP, fibrinogen and E-selectin); Increased: e.g. IL-6 (6%, 95%CI: 2% to 9%). Short-term exposure to PM_2.5_: Association: inflammation, coagulation and endothelial activation.
[73]	Netherlands	Prospective cohort	750	Air pollutants (PM_2.5_, NO_2_, black smoke, SO_2_)	Long-term exposure	CIMT; PWV; AIx	PM_2.5_ (per 5 μg/m^3^ increase): CIMT increased by 0.94% (95% CI: -.2.59% to 4.47%); PWV increased by 0.64% (95% CI: -4.71% to 6.01%); AIx increased by 10.17% (95% CI: -37.82% to 58.17%);
[74]	USA	Cohort study	3996	PM_2.5_, PM_10_	Long-term exposure	radial artery pulse wave and carotid artery ultrasound	Long-term particle mass exposure: Not appear to be associated with greater arterial stiffness.
[75]	Australian	Cross-sectional	606	Ambient PM_2.5_, NO_2_	Long-term exposure	CCS	PM_2.5_ (per μg/m^3^ increase): Association: CCS (≥ 100): (OR 1.20, 95% CI: 1.02 to 1.43); CCS (≥ 400): (OR 1.55, 95% CI: 1.05 to 2.29).
[76]	Germany	Cross-sectional	4291	Ambient PM_2.5_, PM_10_	Long-term exposure	Arterial blood pressure (BP)	Per IQR of PM_2.5_ (2.4 μg/m^3^): Systolic BP increased by 1.4 mmHg (95% CI: 0.5 to 2.3); Diastolic BP increased by 0.9 mmHg (95% CI: 0.4 to 1.4).
[77]	Switzerland	Cross-sectional	1503	Ambient PM_10_, PM_2.5_, UFP	Long-term exposure	CIMT	Vehicular source of PM_2.5_: CIMT increased by 1.67% (95% CI: -0.30 to 3.47%).
[78]	USA	Cross-sectional	6256	Ambient PM_2.5_ (EC, OC, silicon, and sulfur)	Long-term exposure	CIMT, PM_2.5_ components EC, OC, silicon, and sulfur	Per IQR increase of PM_2.5_: Association/increase: CIMT PM_2.5_ (14.7 μm, 95% CI: 9.0 to 20.5); OC (35.1 μm, 95% CI: 26.8 to 43.3); EC (9.6 μm, 95% CI: 3.6 to 15.7); Sulfur (22.7 μm, 95% CI: 15.0 to 30.4).
[79]	Seoul, Korea	Cohort study	364	Ambient PM_2.5_	Long-term exposure	Coronary computed tomographic angiography	PM_2.5_ (per 1 μg/m^3^ increase): Increase/association: HRP (aHR 1.62, 95% CI: 1.22 to 2.15, p < 0.001); fibrofatty and necrotic core component (aHR 1.41, 95% CI: 1.23 to 1.61, p < 0.001); total plaque volume progression (aHR 1.14, 95% CI: 1.05 to 1.23, p = 0.002).
[80]	USA	Cross-sectional	417	Ambient PM_2.5_,O_3_	Long-term exposure	CIMT	PM_2.5_ (per 1 μg/m^3^ increase): CIMT increased by 4.28 μm/y (95% CI: 0.02 to 8.54μm/y).
[81]	Germany	Prospective cohort	4494	Traffic and PM_2.5_	Long-term exposure	CAC	Possible association between PM_2.5_ exposure and CAC
[82]	USA	Cohort study	3506	Ambient PM_2.5_	Long-term exposure	TAC, AAC	No consistent associations between PM_2.5_ and TAC, AAC
[83]	Taiwan	Prospective cohort	30034	Ambient PM_2.5_	Long-term exposure	CRP	PM_2.5_ (per 5 μg/m^3^ increase): Association: systemic inflammation CRP increased by 1.31% (95% CI: 1.00% to 1.63%)
[84]	North Carolina	Cross-sectional	861	PM_10_, PM_2.5_, NO_2_, O_3_	-	CIMT	No associations between PM_2.5_ and CIMT.
[85]	Detroit, MI; Oakland, CA; Pittsburgh, PA; Chicago, IL; and Newark, NJ	Cohort study	1188	PM_2.5_, O_3_	Long-term exposure	CIMT, IAD, plaque presence and plaque index	PM_2.5_ (1 μg/m^3^ higher 5-year mean): CIMT increased 8 μm (95% CI: 1.0 to 15.1), adjusting for cardiovascular disease risk factors; No significant associations between PM_2.5_ and IAD; No associations between PM_2.5_ and plaque presence or plaque index.
[86]	German	Cohort study	4814	PM_2.5_, PM_10_	Long-term exposure	CIMT	PM_2.5_ (interdecile range increase 4.2μg/m^3^): CIMT increased 4.3% (95% CI: 1.9% to 6.7%); PM_10_ (interdecile range increase 6.7μg/m^3^): CIMT increased 1.7% (95% CI: -0.7% to 4.1%).
[87]	Sichuan, China	Longitudinal study	205	Household air pollution (PM_2.5_ and BC)	Short-term exposure (48 h)	BP, PP, cfPWV, AIx	PM_2.5_ (1-ln (μg/m^3^) increase): Association: SBP; PP; cfPWV (-0.1 m/s, 95% CI -0.4 to 0.2) with no difference; slightly higher AIx (1.1%, 95% CI -0.2 to 2.4).
[88]	Puno, Peru	Cross-sectional	266	Householdbiomass fuel	long-term exposure	Measure 24 h indoor PM_2.5_, CIMT, Carotid plaque, BP	Biomass fuel exposure: Increased: CIMT (0.66 vs 0,60 mm, p < 0.001); carotid plaque prevalence (26% vs 14%, p < 0.05); systolic BP (118 vs 111 mm Hg, p < 0.001); median household PM_2.5_ (280 vs 14 μg/m^3^, p < 0.001).
[39]	Taiwan, China	Prospective panel atudy	117	Ambient PM_2.5_, NO_2_	-	baPWV, hsCRP	PM_2.5_ (10 μg/m^3^ increases at 1 day lag): Association: baPWV (2.1%, 95% CI: 0.7%-3.6%; 2.4%, 95% CI: 0.8%-4.0%); No significant association between NO_2_ and baPWV.
[89]	USA	Cross-sectional	798	PM_2.5_	long-term exposure	CIMT	PM_2.5_ (10 μg/m^3^ increases): CIMT increased (5.9%, 95% CI: 1 to 11%); Adjustment of age, never smokers, ≥ 60 years of age women: the strongest associations with CIMT increased (15.7%, 95% CI: 5.7 to 26.6%).
[90]	USA	Cross-sectional	1147	PM_2.5_	long-term exposure	calcium scores	PM_2.5_ (10 μg/m^3^): Aortic calcification (RR=1.06; 95% CI: 0.96 to 1.16); Long-term residence near a PM_2.5_ monitor (RR=1.10; 95% CI: 1.00 to 1.22).
[91]	USA	Cohort study	5172	PM_2.5_	long-term exposure	CIMT	PM_2.5_ (12.5 μg/m^3^ increases): CIMT increased 1 to 3%.
[81]	Geman	Prospective cohort study	4494	PM_2.5_	long-term exposure	CAC	PM_2.5_ (3.91 μg/m^3^): CAC higher 17.2% (95% CI: -5.6 to 45.5%).
[92]	Hebei, China	Cross-sectional	752	Indoor PM_2.5_, CO, SO_2_	Long-term exposure	CIMT, IL-8, CRP, TNF-α, SAA1	Smoky coal combustion-derived indoor air pollutants: Increased: systemic inflammation; The risk of carotid atherosclerosis RR = 1.434 (95% CI: 1.063 to 1.934, p = 0.018).

Note: Short-term exposure means the period of exposure is less than 3 months; Long-term exposure means the period of exposure is longer than 3 months

*AAC* abdominal aortic calcium agatston score, *aHR* adjusted hazard ratio, *AIx* augmentation index, *BC* black carbon, *BP* Blood pressure, *CAC* coronary artery calcification, *CCS* Coronary artery calcium score, *cfPWV* carotid-femoral PWV, *CI* confidence interval, *CIMT* carotid intima-media thickness, *CRP* C-reactive protein, *EC* elemental carbon, *HDL-P* high-density lipoprotein cholesterol particle matter, *HOI* HDL oxidant index, *HDL-C* high-density lipoprotein cholesterol, *HRP* high-risk plaque, *IAD* inter-adventitial diameter, *IMT* intima-media thickness, *IL* interleukin, *O*_*3*_ ozone, *IQR* interquartile, *NO* nitrogen dioxide, *OC* organic carbon, *Ox-LDL* oxidized low-density lipoprotein, *OR* odds ratio, *PM*_*2.5abs*_ absorbance levels of PM_2.5_, *PNacc* particle number of accumulation mode particles, *PP* pilse pressure, *UFP* ultrafine particles (< 0.1μm), *TAC* thoracic aortic calcium agatston score, *SBP* systolic blood pressure, *sCD36* soluble cluster of differentiation 36, *sICAM-1* soluble Intercellular Adhesion Molecule-1, *SO*_*2*_ sulfur dioxide, *PWV* Pulse wave velocity, *baPWV* brachial-ankle pulse wave velocity

**Table 2:** Animal studies on the association between PM_2.5_ exposure and atherosclerosis

Reference	PM_2.5_ source	Mouse model	Diet	Exposure Time	Findings
[94]	Shanghai, China Ambient PM_2.5_	ApoE^-/-^ mice	Normal chow; High-fat diet	8 h/day, 7 days/week, 16 weeks	PM_2.5_ exposure induced and promoted atherosclerotic lesions with significant difference. **Increased:** Atherosclerotic plaque; lipids (ApoB, LDL-C, T-CHO, TG); CD36; ox-LDL; inflammatory cytokines (IL-1β, IL-18); NLRP3, caspase-1, ASC, pro-caspase-1, cleaved-caspase-1; **Decreased:** Lipids (ApoA1 and HDL-C)
[95]	Nanjing, China Ambient PM_2.5_	ApoE^-/-^ mice	High-fat diet	12 weeks	PM_2.5_ exposure amplified atherosclerotic lesions with significant difference. **Increased:** Atherosclerotic plaque; lipid accumulation; TC; LDL-C; Inflammatory cytokines (IL-6, TNF-α); **Deceased:** Anti-inflammatory cytokines (IL-10, TGF-β); CD4^+^CD25^+^Foxp3^+^Tregs; Foxp3
[96]	Beijing, China Ambient PM (PM_2.5_ and PM_10_)	ApoE^-/-^ mice	High-fat diet	24 h/day, 7 days/week, 2 months	PM_2.5_ increased atherosclerotic plaque with significant difference. **Increased:** Lesion area; TC; LDL; ox-LDL; visfatin; systemic inflammation and pulmonary inflammation response (IL-6, TNF-α); MDA **Decreased:** SOD; GSH-Px
[97]	Beijing, China Ambient PM (PM_2.5_ and PM_10_)	ApoE^-/-^ mice	High-fat diet	24 h/day, 7 days/week, 2 months	PM_2.5_ exposure increased atherosclerotic plaque with significant difference. **Increased:** Plaque area; TC; LDL; ox-LDL; systemic inflammation (Hs-CRP, IL-6, TNF-α) and pulmonary inflammation response (IL-6, TNF-α); **Decreased:** T-AOC; SOD
[12]	Michigan State University, USA Ambient PM_2.5_	ApoE^-/-^ or LDLR^-/-^ mice	High-fat diet	6 h/day, 5 days/week, 6 months	PM_2.5_ exposure increased atherosclerotic plaque with significant difference. **Increased:** Lesion area; lipid and collagen content; 7-KCh and uptake; CD36; foam cell formation
[51]	Nanjing, China Ambient PM_2.5_	ApoE^-/-^ mice	High-fat diet	twice/week, 12 weeks or 24 weeks	PM_2.5_ exposure promoted atherosclerotic plaque development and increased plaque vulnerability, with significant difference. **Increased:** Lesion area, lipid; broken aortic elastic fibers; **Decreased:** Collagen content; fibrous cap
[6]	Beijing, China Ambient PM_2.5_	ApoE^-/-^ mice	High-fat diet	Every 3 days, 2 months,	PM_2.5_ exposure increased the formation of atherosclerosis and the influence probably persisted after 1-month recovery, with significant difference. **Increased:** Atherosclerotic lesion; inflammatory cytokines; lipid metabolism alteration.
[98]	Tianjin, China Traffic related PM_2.5_, simulated PM_2.5_	ApoE^-/-^ mice	High-fat diet	Every two days, 10 weeks	Traffic related and simulated PM_2.5_ promoted the formation of atherosclerosis with significant difference. **Increased:** Plaque; T-CHO; LDL-C; TG; MDA; **Decreased:** HDL-C; SOD; GSH-Px
[99]	Northeastern, China Ambient PM_2.5_, WDE, DEG	ApoE^-/-^ mice	Normal chow	average of 5.2 hours/day, 4 days/week, 3 months and 5 months	Exposure to PM_2.5_ for 5 months induced atherosclerotic plaques with significant difference. For plaque exacerbation, PM_2.5_ > WDE > DEG = FA **Increased:** Plaque; vasomotor dysfunction; inflammation
[100]	Yinchuan, China coal-fired PM_2.5_	C57BL/6J mice and ApoE^-/-^ mice	High-fat diet	3 h/day, 1 day/week, 8 weeks	Coal-fired PM_2.5_ significantly promoted the formation atherosclerosis with significant difference. **Increased:** Plaque; foam cells; fibrous cap formation; ET-1; ICAM-1; E-selectin **Decreased:** vWF
[101]	Manhattan, USA PM_2.5_	ApoE^-/-^ mice	Normal chow and High-fat diet	6 h/day, 5 day/week, 6 months	In high-fat diet group, PM_2.5_ increased plaque area compared with FA (p < 0.01); In normal chow group, PM_2.5_ increased plaque area compared with FA (p < 0.15). **Increased:** Plaque area; Cholesterol; Constriction response; CD68; 3-Nitrotyrosine; eNOS; iNOS; **Decreased:** Relaxation response
[102]	Los Angeles freeway, USA PM_2.5_	ApoE^-/-^ mice	regular diet	5 h/day, 3 day/week, 75 hours	PM_2.5_ resulted in aortic atherosclerotic lesion increased trend (p = 0.1). **Increased:** Plaque area; Liver MDA; **Decreased:** HDL anti-inflammatory properties
[103]	New York; USA PM_2.5_	C57BL/6, ApoE^-/-^ mice, ApoE and LDLR double knockout (DK)	High-fat diet and regular diet	6 h/day, 5 day/week, 5 months	PM_2.5_ exposure increased atherosclerotic lesion in ApoE^-/-^ mice (p < 0.05). Atherosclerotic lesion 57% increase in ApoE^-/-^ mice; Atherosclerotic lesion 10% increase in male DK mice and 8% decrease in female DK mice.
[104]	New York; USA PM_2.5_	ApoE^-/-^ mice	High-fat diet	30 mg/kg/day, 8 weeks	PM_2.5_ contributed to the progression of atherosclerosis (p < 0.05). **Increased:** Atherosclerotic plaques; numbers of lesion macrophages; endothelial layer injury; platelets and leukocytes adherence; IL-6; TNF-α; iNOS; IL-12; arginase-1; CD206
[26]	DEP, 1650b, NIST, USA	C57BL/6, ApoE^-/-^ mice	Regular chow or high-fat diet	Once a day during 5 days/week, 3-6 weeks	DEP exposure increased atherosclerotic lesion in ApoE^-/-^ mice (p < 0.05). **Increased:** Atherosclerotic plaques; EPC apoptosis; superoxide production; **Decreased:** Neoangiogenesis; EPC migration; Endothelial cell interity
[25]	DEP, SRM-2975, NIST, USA	C57BL/6, ApoE^-/-^ mice	Regular chow or high-fat diet	Twice weekly instillation	DEP exposure increased atherosclerotic lesion in ApoE^-/-^ mice (p < 0.05). **Increased:** Atherosclerotic plaques; Cholesterol; antioxidant genes in the liver

Note: *Apo A1* apolipoprotein A1, *Apo B* apolipoprotein B, *ASC* apoptosis associated speck like protein, *CD36* cluster of differentiation 36, *DEG* diesel exhaust gases, *DEP* Diesel exhaust particles, *ET-1* endothelin-1, *eNOS* endothelial nitric oxide synthase, *FA* filtered air, *Foxp3* forkhead box transcription factor P3, *GSH-Px* glutathione peroxidase, *HDL-C* high density lipoprotein-cholesterol, *Hs-CRP* high sensitive C-reactive protein, *IL* interleukin, *ICAM-1* Intercellular Adhesion Molecule-1, *iNOS* inducible nitric oxide synthase, *7-KCh* 7-ketocholesterol, *LDL-C* low density lipoprotein-cholesterol, *MDA* malondialdehyde, *NIST* National Institute of Standards and Technology, *NLRP3* NOD-like receptor protein 3, *ox-LDL* oxidized low-density lipoprotein, *PM* particulate matter, *SOD* superoxide dismutase, *T-AOC* total antioxidant capacity, *T-CHO* total cholesterol, *TG* triglycerides, *TGF-β* transforming growth factor-β, *TNF-α* tumor necrosis factor α, *vWF* von willebrand factor, *WDE* whole diesel exhaust

## Ambient PM_2.5_ induces endothelial dysfunction

The endothelial cell monolayers of blood vessels play a role in exchanging macromolecules between the blood and tissues. Mechanical stimuli such as shear stress, inflammatory cytokines and angiotensin-II (Ang-II) affect endothelial permeability [108, 109]. We concentrated on studies indicating that PM_2.5_ impacts vascular endothelial dysfunction. Many studies have demonstrated that PM_2.5_ increases the vascular permeability, impairs endothelial vasomotor function and vascular reparative capacity via different mechanisms and occurs before vascular diseases such as atherosclerosis. Traffic-derived emissions are a major source of ambient PM_2.5_; therefore, literature on the toxicity of traffic emissions (mainly DEPs) on blood vessels and endothelial cells and the similarities between PM_2.5_ and DEPs are also discussed in this review. The effects of PM_2.5_ and DEPs on endothelial cells are shown in Table 3. In brief, the existing evidence shows that PM_2.5_ and DEPs both consistently induces endothelial cytotoxicity through similar mechanisms, such as by increasing endothelial cellular apoptosis via oxidative stress or autophagy, reducing the migration of endothelial cells and enhancing vascular endothelial permeability [111, 117, 118, 122, 123]. The detailed mechanisms of endothelial cytotoxicity induced by PM_2.5_ or traffic-derived pollutants are discussed below. In addition, coal-fired PM_2.5_ also decreases endothelial viability; however, the detailed mechanisms are limited to increases in DNA methylation and oxidative DNA damage in EA.hy926 cells [128].

**Table 3:** PM_2.5_ exposure in endothelial cells

Reference	Endothelial cells lines	PM_2.5_ source	Exposure concentration	PM_2.5_ Exposure time	Evaluation
[110]	EA.hy926	Beijing, China Urban PM_2.5_	PM_2.5_: 2, 10, 40, 100, 200, 1000 μg /cm^2^; SOD (ROS scavenger): 0.5 mg/ml.	24 h	Trace elements in PM_2.5_ suspension, water-insoluble and water-soluble; Cell viability; ROS; MMP; Apoptosis.
[111]	EA.hy926, HUVECs	PM_2.5_ SRM1648a, NIST, USA	PM_2.5_: 1.25, 2.5, 5, 10, 20, 40 μg /cm^2^; 4-PBA (ER stress inhibitor): 1 mM; 3-MA (a classical PI3K III inhibitor, autophagy antagonist): 2.5 mM; Rapa (an mTOR inhibitor, autophagy agonist): 50 nM; Bafi A1 (a proton-pump inhibitor, autolysosome inhibitor): 20 nM.	24 h	Cell viability; ER stress; Autophagy; Apoptosis; Autophagic flux.
[112]	EA.hy926, HUVECs	PM_2.5_ SRM1648a, NIST, USA	PM_2.5_: 1.25, 2.5, 5, 10, 20, 40 μg/cm^2^; Fer-1 and DFOM (ferroptosis inhibitors): 500 nM and 5 μM, respectively.	24 h or 12 h	Cell viability; intracellular iron content; GSH; lipid peroxidation; redox imbalance; ferroptosis-related genes or biomarkers.
[113]	PCAECs	Fine dust, ERM-CZ100, Sigma-Aldrich, USA	Fine dust: 1, 3, 10, 30, 100 μg/ml; NAC: 10 mM; Losartan: 10 μM.	48 h or 1, 4, 24 h	SA-β-gal; platelet aggregation; cell proliferation; Oxidative stress; Relaxation; Senescence.
[114]	HUVECs	Beijing, China	PM_2.5_: 2, 20, 100 μg/ml; NAC: 5 mmol/l	6, 12, 24 h	VE-cadherin; VEGFR2 and MAPK/ERK signaling; ROS; SOD.
[108]	HUVECs	Beijing, China	PM_2.5_: 80 μg/ml; miR-21 inhibitor	24 h	miR-21; target genes; VE-cadherin.
[115]	HUVECs	Mexico	PM_2.5_: 20 μg/cm^2^;	3, 24, 48, 72 h	Oxidative stress; NF-κB; Apoptosis.
[116]	HUVECs	Wuhan, China	PM_2.5_: 6.25, 12.5, 25 μg/ml; SP600125 (JNKs inhibitor); SB203580 (p38K inhibitor); PD98059 (ERKs inhibitor);	24 h	AP-1; Oxidative stress; pro-inflammatory response.
[117]	HUVECs, HMEC-1	PM_2.5_ NIST, USA	PM_2.5_: 100, 200, 400, 800 μg/ml;	24 h	Cell viability; Apoptosis; Migration; Tube formation; ROS; Inflammation.
[118]	EA.hy926	Yuquan Road, Beijing, China	PM_2.5_: 25, 50, 100, 200 μg/ml; SP600125 (JNK inhibitor): 25 μM; U0126 (ERK inhibitor): 10μM; SB203580 (p38 MAPK inhibitor): 25μM; LY294002 (PI3K/AKT inhibitor): 25μM; BAY11-7082 (NF-kB inhibitor): 5μM.	1, 3, 6, 12, 24 h	Cell viability; ROS; Adhesion molecule; Adhesion experiment.
[119]	HUVECs	COFs-derived PM_2.5_	PM_2.5_: 12.5, 25, 50, 75, 100, 200μg/ml; SU5416 (a VEGFR2 inhibitor): 0.5, 1, 2.5, 5, 7.5, 10, 20 μM.	12, 24, 36 h	Cell viability; Tube formation.
[120]	HUVECs	Taiyuan, China	PM_2.5_: 1, 5, 10 μg/ml; Pam3CSK4 (TLR2 agonist): 1μg/ml; LPS (TLR4 agonist): 500μg/ml; anti-TLR2 (TLR2 inhibitor): 10μg/ml; TAK242 (TLR4 inhibitor): 5μmol/l.	12 h	Inflammation.
[121]	MAECs	Wuhan, China	PM_2.5_: 25, 50, 100 μg/ml; NS-398 (COX-2 inhibitor): 10μM.	12, 24 h	Apoptosis.
[122]	HUVECs, ATG12-KO HUVECs	Diesel exhaust particles (DEP)	DEP: 25, 50 μg/ml; NAC: 5 mM; Nutllin-3a: 5 μM; PMA (ROS inducer): 1μM	2, 4, 8, 24 h	Cell viability; ROS; Cytokeleton; Lysosome; Apoptosis; DNA damage; Tube formation; Migration; Autophagy.
[123]	HAECs	DEP	DEP: 12.5, 25, 50μg/ml;	2, 4, 6 h	Permeability; LDH; Apoptosis; ZO-1.
[124]	HUVECs	Non-industry district, Shanghai, China	PM_2.5_: 100, 200, 400 μg/ml; Atorvastatin: 0.1, 1, 10 μmol/l.	24 h	Water-soluble and organic extracts; Cell viability; Oxidative stress; Cytokines.
[125]	HCAECs	Southern Taiwan	PM_2.5_: 20, 50 μg/ml;	4 h	Metal fume particles; Cell viability; 8-OHdG; IL-6; NO.
[126]	HUVECs	Mexico	PM_2.5_: 5, 10, 20, 40 μg/cm^2^; TNF-α: 10 ng/ml.	6 or 24 h	Cell viability; Adhesion; Adhesion molecules.
[127]	HUVECs	Beijing, China	PM_2.5_: 5, 25, 50, 100, 200 μg/ml; Rap: 100 nmol/l; 3-MA: 5 mmol/l.	24 h	Cell viability; Autophagosome; Autophagy.
[128]	EA.hy926	Coal-fired PM_2.5_ (Yinchuan, Datong, Jingxi, Zhijin, China)	PM_2.5_: 10, 25, 50 μg/ml;	24 h	Cell viability; DNA methylation; DNA damage.

Note: *AP-1* activation protein-1, *Bafi A1* Bafilomycin A1, *DEP* Diesel exhaust particles, *DFOM* Deferoamine mesylate, *EA.hy926* human umbilical vein cell line, *ER* endoplasmic reticulum, *ERK* extracellular signal-regulated kinase, *Fer-1* Ferrostatin-1, *GSH* glutamate, *HCAECs* human coronary artery endothelial cells, *HMEC-1* human microvascular endothelial cells, *IL* interleukin, *LDH* lactate dehydrogenase, *MAECs* Mouse aorta endothelial cells, *3-MA* 3-Methyadenine, *MAPK* mitogen-activated protein kinase, *MMP* Mitochondrial membrane potential, *NAC* N-acetyl-L-cysteine, *NF-κB* nuclear factor kappa-B, *NIST* National Institute of Standards and Technology, *NO* nitric oxide, *8-OHdG* 8-hydroxy-2’-deoxyguanosine, *PAH* polycyclic aromatic hydrocarbon, *4-PBA* 4-phenylbutyrate, *PMA* Phorbol-myristate-acetate, *Rapa/Rap* Rapamycin, *ROS* reactive oxygen species, *SA-β-gal* Senescence-associated (beta)-galactosidase, *TNF-α* tumor necrosis factor α, *VE-cadherin* vascular endothelial cadherin, *VEGFR2* vascular endothelial growth factor receptor 2, *VOC* volatile organic compounds, *ZO-1* Zonular Occludin-1

### Ambient PM_2.5_ increases vascular endothelial permeability

**Fig. 2:**
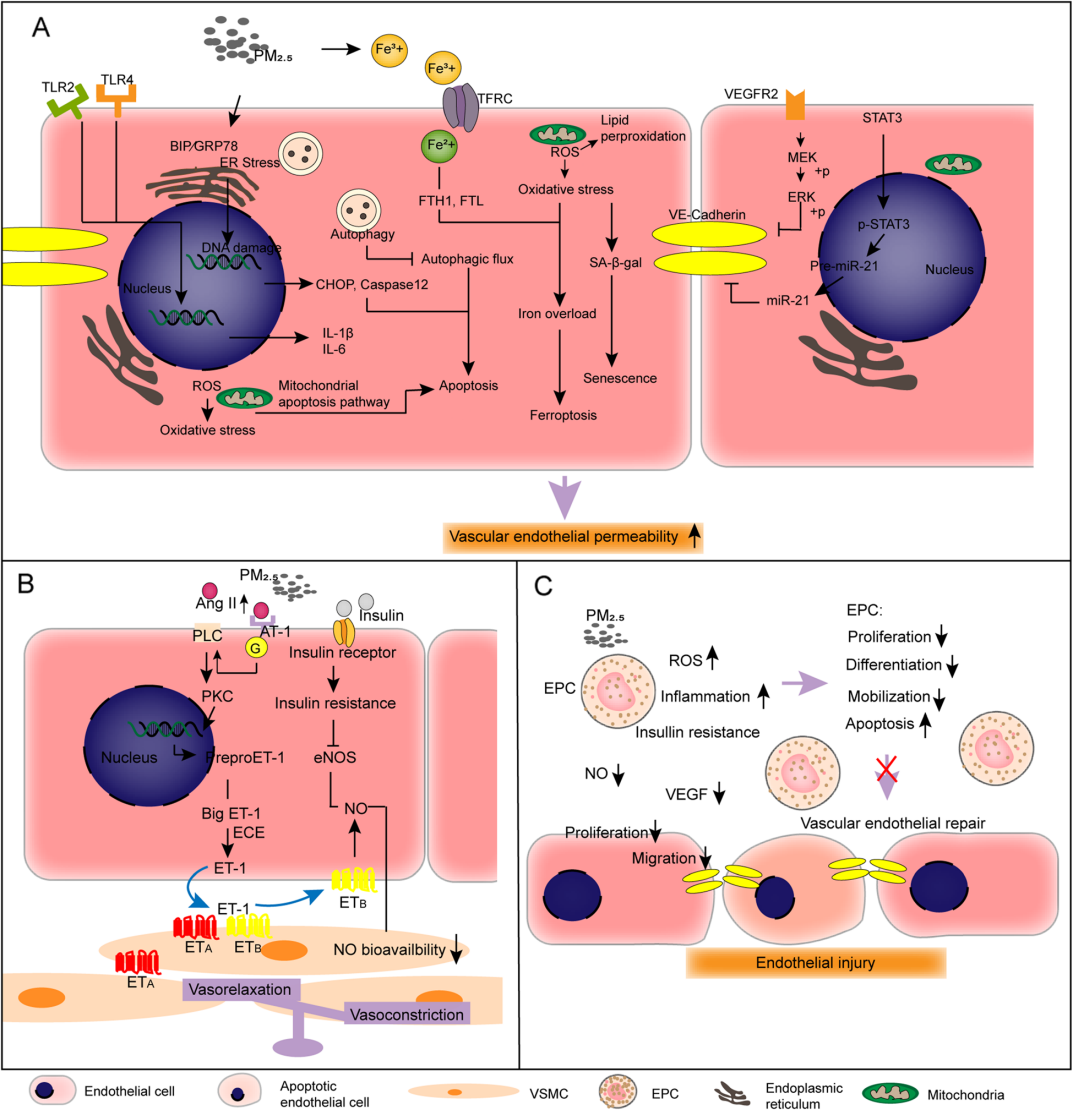
Summary of the main mechanisms of PM_2.5_-caused vascular endothelial injury. Mainly involved three aspects: **a**. PM_2.5_ increased vascular endothelial permeability; **b**. PM_2.5_ impaired vasomotor function; **c**. PM_2.5_ declined vascular reparative capacity

The proposed mechanism of PM_2.5_-triggered vascular endothelial permeability increase is presented in Fig. 2a. In Balb/c mice exposed to low-dose (1.27 mg/kg) and high-dose (6.34 mg/kg) PM_2.5_ through the tail vein for 48 h, PM_2.5_ destroyed the integrity of vessels, as assessed by the Evans blue infiltration assay, and the results confirmed that PM_2.5_ increased vascular permeability *in vivo* [114]. DEPs increase vascular endothelial permeability by downregulating the expression of zonula occludens-1 (ZO-1, a tight junction protein) [123]. Ambient PM_2.5_ exposure disrupts the balance between antioxidation and oxidation in vascular endothelial cells, leading to increased permeability of the endothelial monolayer. Epidemiological evidence has shown that exposure to PM_2.5_ reduces the anti-inflammatory and antioxidant capacity of high-density lipoprotein (HDL), and decreases the expression of antioxidant markers such as glutathione peroxidase (GSH) and superoxide dismutase (SOD) [67, 129]. A review showed that particulate matter impairs HDL function via oxidative pathways [53]. Importantly, evidence has shown that exercise training enhances HDL functions, including cholesterol efflux capacity and antioxidant capacity, and protects against endothelial dysfunction induced by PM_2.5_ [130]. PM_2.5_ decreases the mitochondrial membrane potential, increases reactive oxygen species (ROS) generation, and causes oxidative stress, inflammation and apoptosis in EA.hy926 cells and human umbilical vein endothelial cells (HUVECs) [110, 115, 124]. ROS play important roles in inflammatory responses, apoptosis, and cell growth, as well as in the oxidation of LDL cholesterol [131]. PM_2.5_ induces ROS generation and endothelial cell apoptosis through the mitochondrial pathway in EA.hy926 cells [132]. PM_2.5_ induces cell autophagy and apoptosis via endoplasmic reticulum (ER) stress in EA.hy926 cells and HUVECs [111]. Although normal autophagy seems to protect cells from PM_2.5_-triggered apoptosis, PM_2.5_ blocks autophagic flux and then robustly aggravates endothelial cell apoptosis [111, 122, 127]. Effective inhibition of ER stress using 4-PBA (an ER stress inhibitor) contributes to the alleviation of PM_2.5_ induced cell apoptosis and the expression of LC3II [111]. Bafi A1 (an autolysosome inhibitor) aggravates PM_2.5_-induced cell apoptosis by disrupting autophagic flux [111]. Exposure to PM_2.5_ induces activation of the inflammatory cyclooxygenase-2 (COX-2)/prostaglandin E synthase (PGES)/prostaglandin E 2 (PGE2) axis and promotes the inflammatory response and apoptosis in mouse aorta endothelial cells (MAECs) [121]. Excessive apoptosis triggers an increase in transcellular permeability in the vascular endothelial monolayer [133]. Ambient PM_2.5_ disrupts iron uptake and storage by regulating the expression of transferrin receptor (TFRC), ferritin light chain (FTL) and heavy chain (FTH1), causing intracellular iron overload and subsequently provoking ferroptosis in EA.hy926 cells and HUVECs [112]. PM_2.5_ induces ROS production and lipid peroxidation in endothelial cells and increases membrane permeability [112, 134]. Furthermore, PM_2.5_ induces senescence associated-β galactosidase (SA-β-gal) activation via redox sensitivity of the local angiotensin system in premature coronary arterial endothelial cells (PCAECs), leading to endothelial senescence [113]. The presence of senescent endothelial cells in a nonsenescent monolayer disrupts the tight junction morphology of surrounding young cells and increases the permeability of the monolayer [135].

Vascular endothelial (VE)-cadherin is largely expressed on endothelial cell membranes. The extracellular domain of VE-cadherin mediates endothelial cell-to-cell adhesion through hemophilic trans interactions, whereas its cytoplasmic tail associates with the actin cytoskeleton, strengthening the adhesion junction between endothelial cells [136]. Thus, VE-cadherin plays a key role in maintaining endothelial barrier integrity, controlling the transmembrane movement of macromolecule substances such as blood cells. PM_2.5_ induces the phosphorylation of vascular endothelial growth factor receptor 2 (VEGFR2) on the endothelial cell membrane and activates downstream mitogen-activated protein kinase (MAPK)/extracellular signal-regulated kinase (ERK) signaling, leading to the shedding of adhesion connexin VE-cadherin [114]. PM_2.5_ induces endothelial cell cytoskeleton rearrangement via Rho-dependent pathways to facilitate vascular hyperpermeability [137]. Targeting of tissue inhibitor of metalloproteinase 3 (TIMP3)/MMP9 and VE-cadherin by miR-21 in response to PM_2.5_ increases vascular endothelial cell permeability in HUVECs [108]. Inflammation stimulates a series of signaling pathways that reduce the level of VE-cadherin expressed or induces VE-cadherin phosphorylation and then destroys the adhesion structure [138].

### Ambient PM_2.5_ impairs vasomotor function

Endothelin-1 (ET-1) is a protein primarily produced by endothelial cells that regulates cell proliferation and vascular tone by activating its receptors, including type A (ET_A_) and type B (ET_B_) [139]. Evidence has indicated that inflammation, ischemia and hypoxia stimulate the expression of ET-1 and its receptors [140]. Qinghua Sun et al. exposed ApoE^-/-^ mice to concentrated ambient PM_2.5_ for 6 months and assessed the vasoconstriction of aortic rings in response to phenylephrine and serotonin and vasorelaxation in response to acetylcholine. The results demonstrated that PM_2.5_ significantly increased the constriction of the aorta, especially in high-fat diet-fed mice [101]. The proposed mechanism of by which PM_2.5_ causes endothelial vasomotor function impairment is presented in Fig. 2b. PM_2.5_ elevates the circulating levels of AngII, locally activates the AngII/AngII type 1 receptor (AT1R) axis and activates phospholipase C (PLC) and protein kinase C (PKC), promoting ET-1 biosynthesis in HUVECs [116]. ET-1 is released from endothelial cells, acts on the endothelial ET_B_ receptor and increases nitric oxide (NO) production [141]. The production of NO by endothelial cells contributes to regulating vasomotor tone. NO cats on circulating blood platelets, leukocytes and adjacent smooth muscle cells and reduces smooth muscle cell contractility [142]. PM_2.5_ impairs the balance of vasorelaxation by oxidative stress, and superoxide radicals combine with NO to form peroxynitrite, thus reducing NO bioavailability in the vessel wall [143]. Furthermore, PM_2.5_ upregulates the expression of ET_B_ and ET_A_ receptors in rat coronary arteries [144]. ET_B_ in vascular endothelial cells mediates the vasodilation, while ET_A_ and ET_B_ in vascular smooth muscle cells mediate the contractility, especially ET_A_ activation, which plays a greater role in coronary vasoconstriction [141]. ET-1 in the vasculature causes brief vessel relaxation due to ET_B_ activation in endothelial cells. However, this effect is quickly reversed by ET-1 binding to ET_A_, which reduces NO production in vascular smooth muscle cells and leads to the well-known constrictive effects of ET-1 in the vasculature [145]. Therefore, PM_2.5_ upregulates the expression of ET_B_ and ET_A_ receptors in coronary arteries, but PM_2.5_ mainly increases vasoconstriction and contributes to the progression of atherosclerosis. AngII enhances ET-1-mediated vasoconstriction by upregulating the expression of ET_A_ in VSMCs [146]. In addition, PM_2.5_ exposure causes vascular insulin resistance and suppresses insulin-stimulated endothelial nitric oxide synthase (eNOS) phosphorylation (likely an endothelial-specific event) [147]. Insulin stimulates the phosphorylation of eNOS and increases eNOS activity and NO production [148–150]. Therefore, PM_2.5_-provoked endothelial insulin resistance could be a key event in regulating vascular tone. In brief, PM_2.5_ shifts the balance of vasomotor tone towards vasoconstriction by increasing the levels of ET-1 and its receptors, as well as decreasing NO production and bioavailability. Exercise training effectively prevents the imbalance in vasomotor function triggered by PM_2.5_ [130].

### Ambient PM_2.5_ suppresses vascular endothelial repair

Endothelial progenitor cells (EPCs), a group of stem cells/progenitor cells, settle in the adult bone marrow and can mobilize to the peripheral blood, home to sites of vascular injury, proliferate and differentiate into endothelial cells, and facilitate vascular recovery [151]. In addition to exerting antioxidative and anti-inflammatory effects, HDL protects EPCs by increasing eNOS levels and decreasing MMP9 levels, thereby reducing the apoptosis of EPCs [152]. Bone marrow-derived EPCs from C57BL/6 mice exposed to PM_2.5_ inhalation for 9 or 30 days were injected into unexposed mice subjected to hind limb ischemia and vascular perfusion was assessed by laser Doppler perfusion imaging (LDPI). The results confirmed that PM_2.5_ significantly impaired angiogenesis and that bone marrow-derived EPCs have vascular reparative capacity *in vivo* [153]. The proposed mechanism of PM_2.5_-triggered vascular repair suppression is presented in Fig. 2c. In C57BL/6 mice exposed to concentrated PM_2.5_ inhalation for 9 or 30 consecutive days (6 h/day), PM_2.5_ impaired endothelial progenitor cellular differentiation and mobilization through vascular insulin resistance and nuclear factor kappa-B (NF-κB) and inflammasome activation, while insulin sensitizers prevented PM_2.5_-triggered vascular insulin resistance and inflammation and decreased circulating EPCs [154]. ROS and inflammation suppress the proliferation of EPCs and enhance the apoptosis of EPCs [155]. Furthermore, PM_2.5_ decreases the abundance of EPCs, and impairs EPC functions and prevents EPC-mediated vascular endothelial recovery associated with vascular endothelial growth factor (VEGF) resistance and a decrease in NO bioavailability [153]. In addition to EPCs, the proliferation and migration abilities of mature endothelial cells are additional important factors in endothelial injury repair. Endothelial cell proliferation and migration are initially required for arterial repair after injury [156]. PM_2.5_ decreases the viability and suppresses the proliferation and migration of HUVECs and human microvascular endothelial cells through oxidative stress [117]. In brief, PM_2.5_ disrupts two major factors involved in repairing vascular endothelial damage: a. the abundance and function of EPCs and b. the proliferation and migration abilities of endothelial cells.

## Ambient PM_2.5_-triggered endothelial injury and atherosclerosis

### Ambient PM_2.5_ causes a pro-coagulant state

Atherosclerosis is characterized by the accumulation of inflammatory cells and lipids in the walls of arteries. The intact endothelial cell layer exerts the first defense to hinder the development of atherosclerosis. Endothelial cell-derived mediators take part in hemostasis, including tissue factor (TF), tissue factor pathway inhibitors (TFPI), thrombomodulin (TM), and von Willebrand factor (vWF) [157, 158]. For example, normal vascular endothelium-synthesized TFPI regulates the balance between coagulation and fibrinolysis [44]. Exposure to air pollution affects each of these dynamic processes, and increasing evidence suggests that the balance of between platelet activation, coagulation and fibrinolysis shifts towards a pro-coagulant and anti-fibrinolytic state [8]. Sprague-Dawley (SD) rats were exposed to PM_2.5_ (1.8, 5.4, 16.2 mg/kg bw) by intratracheal instillation every three days for 30 days, and the results showed that PM_2.5_ increased the expression of TF in the vessel wall and activated coagulation factors VII and X and the formation of thrombin [158–160]. PM_2.5_ reduces the expression of TM in the vascular endothelial monolayer, thereby decreasing anti-coagulant function [160]. vWF is mainly released by activated endothelial cells, which bridge platelets and aggregates in the injured vessel walls [161]. PM_2.5_ downregulates the expression of vWF in the serum and promotes the adherence of platelets to injured endothelial layers, implying that vWF is consumed during the process of platelet aggregation after exposure to PM_2.5_ [104, 160]. Platelet adhesion, aggregation and coagulation are implicated in inflammatory pathologies of atherosclerosis [162, 163].

### Ambient PM_2.5_ induces an inflammatory response

Short-term exposure to PM_2.5_ induces systemic inflammation and increases the circulation levels of inflammatory biomarkers such as CRP, tumor necrosis factor α (TNF-α), IL-6, IL-8 and MCP-1 [35, 164]. PM_2.5_ triggers the secretion of IL-6 and IL-1β by activating the TLR-mediated pathway in HUVECs, while TLR2 and TLR4 inhibitors reduce the PM_2.5_-triggered inflammatory response [120]. PM_2.5_ triggers endothelial activation, increases the expression of adhesion molecules (ICAM-1 and VCAM-1) and induces THP-1 cell adhesion to endothelial cells through the ERK/AKT/NF-κB-dependent pathway in EA.hy926 cells; moreover, ERK/AKT/NF-κB inhibitors have been used to demonstrate the abovementioned effects [118]. Monocytes in the blood adhere to endothelial cells through adhesion molecules and then migrate into the vascular wall [126, 165]. Monocytes that enter the blood vessel wall transform into macrophages, which clear lipids and dead or dying cells [166]. Exposure of ApoE^-/-^ or LDLR^-/-^ mice to concentrated ambient PM_2.5_ for 6 months (6 h/day, 5 days/week) showed that PM_2.5_ significantly increases the expression of CD36 in plaque macrophages, increases the internalization of ox-LDL and mediates macrophage-derived foam cell formation [12]. Both macrophage-derived foam cells and necrotic cells release various inflammatory factors (such as TNF, IL-1, and IL-6), thereby expanding the inflammatory response cascade and inducing persistent inflammation in local blood vessels [166]. Moreover, evidence from animal experiments has shown that inflammation is significantly unregulated in ApoE^-/-^ mice after exposure to PM_2.5_ (10 mg/kg bw) for two months and that inflammation increases even if PM_2.5_ exposure is stopped [6]. The above evidence focuses on the effects of PM_2.5_ on inflammation and atherosclerosis.

### Ambient PM_2.5_ promotes lipid deposition

Lipid deposition is one of the key factors promoting the development of atherosclerosis, especially ox-LDL deposition, which contributes to necrotic core formation. In the past few decades, lowering lipid levels has been the main strategy for the treatment of atherosclerosis [167]. However, currently, two views about how lipids enter and deposit in the vascular wall are held. It has been believed that damaged vascular walls cause LDL infiltration into the vascular wall and induce the development of atherosclerosis [168]. Professor Shaul holds a different view, suggesting that the receptor scavenger receptor type B1 (SR-B1) on endothelial cells has a transcytosis effect on LDL and promotes the accumulation of LDL in the vascular wall [169]. Lectin-like oxidized low-density lipoprotein receptor-1 (LOX-1) is expressed in endothelial cells, monocytes/macrophages and vascular smooth muscle cells and is essential for binding to oxLDL [170]. Pro-inflammatory and pro-oxidant, and mechanical stimuli including ox-LDL, TNF-α, Ang II and shear stress, can rapidly activate the expression of LOX-1 [171]. In endothelial cells, LOX-1 activation induces inflammation, reduces eNOS activation and NO availability, and triggers endothelial dysfunction [172]. Ox-LDL induces the release of the soluble form of LOX-1 (sLOX-1) from endothelial cells into the circulation and the level of sLOX-1 correlates with carotid plaque inflammation and risk for ischemic stroke [173]. Inhaled vehicle emissions trigger significant increases in plasma sLOX-1 levels in humans and mediate the upregulation of ET-1 and MMP9 expression via ox-LDL-LOX-1 receptor signaling, further inducing vascular effects [174]. LOX-1 protein levels are increased in the aorta after coexposure to ozone and DEPs [175]. Moreover, in ApoE^-/-^ mice exposed to a mixture of gasoline and diesel engine exhaust (MVE), the expression of LOX-1 was increased in cerebral microvascular endothelial cells, and at least in part, MVE altered the structure and integrity of the brain microvasculature via LOX-1 signaling [176]. Many studies have demonstrated that PM_2.5_ contributes to lipid dysregulation in the sera of ApoE^-/-^ mice and promotes macrophage engulfment of ox-LDL through surface scavenger receptors to induce foam cell formation [6, 12, 177]. However, studies on the effect of PM_2.5_ on lipid uptake and transport in endothelial cells are lacking, although limited evidence has shown that traffic-derived pollutants increase LOX-1 signaling in endothelial cells. In addition to inflammation, lipid uptake and transport are key factors in the development of atherosclerosis. Thus, the effect of PM_2.5_ on the binding of ox-LDL to endothelial cells is an area of intense investigation.

## Indoor PM_2.5_ elicits endothelial dysfunction and atherosclerosis

WHO data has shown that more than 41% of households are still using solid fuels and kerosene for cooking, producing harmful smoke in the home and causing the death of approximately 916 thousand individuals from cardiovascular disease [178]. CIMT is a marker of subclinical atherosclerosis. Recently, epidemiological evidence showed that PM_2.5_ emissions from biomass cooking fuel in peri-urban villages of India were positively associated with increased CIMT [61]. Consistent with this study, Matthew S Painschab et al. also showed that indoor PM_2.5_ sourced from biomass fuel in Puno, Peru, was associated with CIMT, an enhanced prevalence of atherosclerotic plaque and increased blood pressure [88]. In the rural villages of Sichuan, China, household PM_2.5_ from biomass stoves is associated with central hemodynamics and increased blood pressure; however, it is not associated with pulse wave velocity (PWV, a marker of arterial stiffness) [87]. Indoor particles impair microvascular function through inflammation and oxidative stress [179]. Cooking oil fumes (COFs), the main pollutants in kitchen air, can significantly reduce cellular viability, and inhibit angiogenesis in HUVECs through the ROS-mediated NLRP3 inflammasome pathway or VEGF/VEGFR2/MEK1/2/ERK1/2/mTOR pathway [40, 119]. COF-derived PM_2.5_ mediates autophagy via the ROS/AKT/mTOR axis in HUVECs [180]. Evidence for the mediating role of indoor PM_2.5_ in vascular endothelial dysfunction is limited.

## Conclusion

In summary, PM_2.5_ exposure is positively associated with atherosclerosis based on epidemiological evidence. Epidemiological and animal experimental evidence has established that PM_2.5_-induced atherosclerosis is mainly mediated by inflammation and lipid metabolism alterations. On the basis of *in vivo* and *in vitro* studies, PM_2.5_ induces vascular endothelial dysfunction and a procoagulant state and increases inflammation and lipid abnormalities, thus promoting the development of atherosclerosis. However, only a few studies have tried to explore preventive measures. It would be meaningful to explore measures or targets that can contribute to the prevention of PM_2.5_-induced endothelial dysfunction or atherosclerosis, which remains to be solved before the environment improves. Changes occur at the molecular level significantly earlier than histopathology and clinical symptoms. Consequently, improving the understanding of molecular mechanisms will be helpful in preventing the occurrence or development of atherosclerosis, or identifying potential therapeutic targets for atherosclerosis treatment.

## Data Availability

Databases/repositories and materials is not applicable in this review.

## Abbreviations

AAC: Abdominal aortic calcium agatston score
ABCA1: ATP-binding cassette transporter A1
AngII: Angiotensin II
aHR: Adjusted hazard ratio
AT1R: AngII type 1 receptor
AIx: Augmentation index
ApoA1: Apolipoprotein A1
ApoB: Apolipoprotein B
AP-1: Activation protein-1
ASC: Apoptosis associated speck like protein
Bafi A1: Bafilomycin A1
BC: Black carbon
BP: Blood pressure
baPWV: Brachial-ankle pulse wave velocity
CAC: Coronary artery calcification
CCS: Coronary artery calcium score
CD36: Cluster of differentiation 36
cfPWV: Carotid-femoral PWV
CI: Confidence interval
CIMT: Carotid intima-media thickness
COFs: Cooking oil fumes
COX-2: Cyclooxygenase -2
CRP: C-reactive protein
DEG: Diesel exhaust gases
DFOM: Deferoamine mesylate
EA.hy926: Human umbilical vein cell line
EC: Elemental carbon
eNOS: Endothelial nitric oxide synthase
EPC: Endothelial progenitor cells
ET-1: Endothelin-1
ET_A_: Endothelin type A
ET_B_: Endothelin type B
ER: Endoplasmic reticulum
ERK: Extracellular signal-regulated kinase
FA: Filtered air
Fer-1: Ferrostatin-1
Foxp3: Forkhead box transcription factor P3
FTL: Ferritin light chain
FTH1: Ferritin heavy chain
GSH: Glutathione peroxidase
GSH-Px: Glutathione peroxidase
HDL: High-density lipoprotein
HDL-C: High density lipoprotein-cholesterol
HDL-P: High-density lipoprotein cholesterol particle matter
HOI: HDL oxidant index
Hs-CRP: High sensitive C-reactive protein
HRP: High-risk plaque
HUVECs: Human umbilical vein endothelial cells
IL: Interleukin
IAD: Inter-adventitial diameter
IMT: Intima-media thickness
iNOS: Inducible nitric oxide synthase
IQR: Interquartile
7-KCh: 7-ketocholesterol
LDH: Lactate dehydrogenase
LDL: Low-density lipoprotein
LDL-C: Low density lipoprotein-cholesterol
LDPI: Laser Doppler perfusion imaging
LOX-1: Lectin-like oxidized low-density lipoprotein receptor-1
LXR-α: Liver X receptor α
MAECs: Mouse aorta endothelial cells
3-MA: 3-Methyadenine
MAPK: Mitogen-activated protein kinase
MCP: Monocyte chemoattractant protein
MDA: Malondialdehyde
MMP: Matrix metalloproteinase
MMP: Mitochondrial membrane potential
MRI: Magnetic resonance imaging
NAC: N-acetyl-L-cysteine
NF-κB: Nuclear factor kappa-B
NO: Nitric oxide
NIST: National Institute of Standards and Technology
8-OHdG: 8-hydroxy-2’-deoxyguanosine
NLRP3: NOD-like receptor protein 3
O_3_: Ozone
OC: Organic carbon
OR: Odds ratio
ox-LDL: Oxidized low-density lipoprotein
PAH: Polycyclic aromatic hydrocarbon
4-PBA: 4-phenylbutyrate
PCAECs: Premature coronary arterial endothelial cells
PGES: Prostaglandin E synthase
PGE2: Prostaglandin E 2
PM_2.5_: Fine particulate matter
PM_2.5abs_: Absorbance levels of PM_2.5_
PMA: Phorbol-myristate-acetate
PLC: Phospholipase C
PKC: Protein kinase C
PNacc: Particle number of accumulation mode particles
PP: Pilse pressure
PWV: Pulse wave velocity
Rapa/Rap: Rapamycin
ROS: Reactive oxygen species
SA-β-gal: Senescence associated-β galactosidase
SBP: Systolic blood pressure
SO_2_: Sulfur dioxide
SOD: Superoxide dismutase
sICAM-1: Soluble Intercellular Adhesion Molecule-1
SR-BI: Scavenger receptor type B1
TAC: Thoracic aortic calcium agatston score
T-AOC: Total antioxidant capacity
TC: Total cholesterol
TF: Tissue factor
TFPI: Tissue factor pathway inhibitors
TFRC: Transferrin receptor
TG: Triglycerides
TGF-β: Ttransforming growth factor-β
TM: Thrombomodulin
TIMP3: Tissue inhibitor of metalloproteinase 3
TNF-α: Tumor necrosis factor α
TLR: Toll-like receptor
UFP: Ultrafine particles
VCAM-1: Vascular cell adhesion molecule-1
VE: Vascular endothelial
VEGF: Vascular endothelial growth factor
VEGFR2: Vascular endothelial growth factor receptor 2
VOC: Volatile organic compounds
VSMCs: Vascular smooth muscle cells
vWF: Von willebrand factor
WDE: Whole diesel exhaust
WHO: World Health Organization
ZO-1: zonula occludens-1
